# Simulation and Assessment of Thermal-Stress Analysis of Welding Materials in IGBT

**DOI:** 10.3390/mi15121519

**Published:** 2024-12-20

**Authors:** Yang Yang, Jibing Chen, Bowen Liu, Yiping Wu

**Affiliations:** 1 School of Mechanical Engineering, Wuhan Polytechnic University, Wuhan 420023, China; 2 School of Material Science and Engineering, Huazhong University of Science & Technology, Wuhan 430074, China

**Keywords:** insulated gate bipolar transistors (IGBT), thermal-stress analysis, temperature distribution, equivalent stress and strain, crack and void damage

## Abstract

Insulated gate bipolar transistors (IGBTs), as an important power semiconductor device, are susceptible to thermal stress, thermal fatigue, and mechanical stresses under high-voltage, high-current, and high-power conditions. Elevated heat dissipation within the module leads to fluctuating rises in temperature that accelerate its own degradation and failure, ultimately causing damage to the module as a whole and posing a threat to operator safety. Through ANSYS Workbench simulation analysis, it is possible to accurately predict the temperature distribution, equivalent stress, and equivalent strain of solder materials under actual working conditions, thus revealing the changing laws of the heat–mechanical interaction in solder materials. Simulation analysis results show that, under steady-state operating conditions, the highest point of the IGBT module’s overall junction temperature occurs in the center of the chip. Nanogold exhibited the best performance in terms of temperature and equivalent stress-strain among the five solders studied in this paper; defects near the edges caused greater harm to the module compared to those closer to the solder layer’s center. In terms of stress, defects located near the edge corners produced larger strains. Crazing damage in joints allows for a faster transfer of heat sources away from the center; in terms of stress, crazing has fewer detrimental effects on the integrity of the module as compared to through cracks. Simulation analysis can model the interaction of heat and equipment under realistic work conditions, comparing and evaluating different types of solder materials to select the most suitable solder material for product design and material selection. This aids in enhancing design precision and reliability.

## 1. Introduction

As the world progresses and electrification levels increase, power systems are evolving towards both high proportions of renewable resources and high percentages of power electronic devices. A myriad of power electronic devices are extensively employed in the source-to-grid-to-load segment of power systems [1]. Among these, power semiconductors serve as the core equipment for energy transmission and conversion, and their prospects are expanding. However, with a failure rate of up to 31%, how to enhance the reliability of power devices has become an important challenge. The IGBT (insulated gate bipolar transistor), hailed as the “CPU” of the power electronics industry, plays a central role as a key component in industrial control and automation [2,3]. It can regulate voltages, currents, frequencies, and phases in circuits according to signal instructions within the device to achieve precise control [4]. Consequently, IGBTs find wide application in the fields of new energy vehicles, motor efficiency improvement, urban rail transit, smart grids, aerospace, household appliances, and automotive electronics [5].

Due to the power module’s long-term operation in an on-off cycle, the losses generated ultimately dissipate as heat energy [6]. When the heat generated by an IGBT power module reaches a certain level, it causes the temperature on the bonding line to soar abruptly, thus resulting in a temperature disparity between contact points and the bonding line. This temperature difference subsequently gives rise to thermal stress, which can severely damage the bonding line and ultimately lead to its fracture or detachment [7,8]. According to the mechanism of failure, Li et al. classify the failure of the power module package for IGBTs into key bonding line failure, weld layer failure, electromigration, electrochemical corrosion, and metallization reconfiguration [9]. Kexin et al. discovered through temperature cycling experiments that the bonding lines tend to fail when subjected to temperature fluctuations greater than 100 K [10]. Ji et al., through simulated experiments mimicking normal operating conditions, found that the distribution of current on the IGBT bonding lines and the different thermal expansion coefficients of different materials cause bond line damage [11]. Kang et al., in a review of the literature, deduced that when the module is in operation, the power loss of the IGBT chip and the joule heat generated by the bonding lines would raise the junction temperature of the bonding lines, leading to a temperature gradient at the contact points and bonding lines, resulting in shear stress [12].

Within the bonded line-type IGBT power module, temperature disparities also exist between the solder layer and its adjacent components. Under the stimulation of cold-hot cycle conditions, the solder layer will crack, which further evolves into solder layer separation. This will further increase the temperature disparity between the solder layer and its adjacent components, leading to an ever-increasing thermal stress until the solder layer completely fails. Luo et al. summarize that the main forms of coating failure are porosity and cracks, which are largely due to the manufacturing process of the solder, making it difficult to avoid. With the cycle of opening and closing, the coating is subjected to thermal stress, causing the porosity to increase [13,14,15]. According to Vermeersch B et al., solder layer damage generally initiates with cracks, which then deepen to form voids [16].

In recent years, research in chip welding has become a hot topic in materials science and electronic packaging. Traditionally, chip solder paste was primarily based on tin–lead alloys; however, the toxicity issue posed by lead led to the proliferation of research efforts towards alternative lead-free solder pastes in line with international eco-regulations [17]. Qian et al. discovered that nanosilver paste outperforms other solder alloys in terms of conductivity and thermal conductivity, making it one of the best solder alloys for overall performance [18,19,20]. However, Abuelnaga et al. noted that its wetting behavior and reliability under high temperatures still require improvement [21]. In addition, in order to enhance the performance of the solder, Zhao et al. employed various methods, such as by incorporating metal nanoparticles, that significantly improved the wetting ability and mechanical strength of the solder [22,23,24].

This paper employs the ANSYS Workbench platform to conduct thermal stress analysis simulations and analyze the solder layer in its operating state [25], observing the thermal-resistance maps, stress-strain diagrams, and deformation patterns of various solder materials. This is carried out with the aim of providing certain data references and support for selecting the most suitable solder material under different conditions, thereby enhancing design accuracy and reliability [26]. In tandem, this paper also compares the eco-friendliness of various solder materials in line with the concept of green development. It selects the best solder coating material by considering the performance characteristics of the solder materials.

## 2. Model Development and Its Associated Parameters

### 2.1. Simulating the Thermal–Hydraulic Coupling of IGBTs

In the thermal-mechanical coupling simulation analysis of the solder layer in an IGBT module, temperature distribution and stress-strain are the focal subjects of the study. To achieve thermal-hydraulic coupled finite element analysis, this paper employs ANSYS Workbench for finite element analysis (FEA). Based on the heat conduction differential equation in thermodynamics, numerical calculations are performed using the matrix form of parameters and boundary conditions corresponding to a three-dimensional model [27]. Firstly, an appropriately sized IGBT 3D model is created using modeling software and then imported into the database of the simulation analysis software. Secondly, the real environment is simulated by setting parameters such as density and thermal conductivity for various materials. Finally, experimental conditions were set up to approximate the IGBT module’s behavior in a normal working environment, ensuring the reliability of the experiment’s conclusions.

### 2.2. Finite Element Model Construction and Parameter Setting

**Figure 1:**
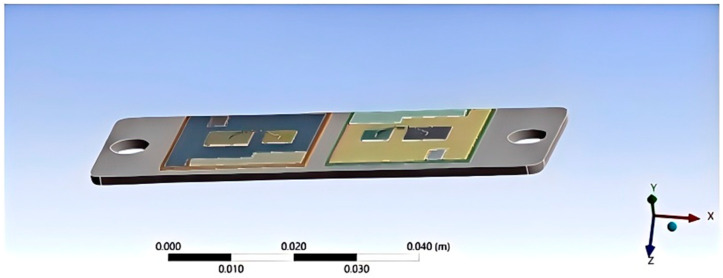
3D geometric model of the IGBT module.

This study employs the geometric model parameters of Infineon’s FF50R12RT4 module. The dimensions were obtained through reference to manuals and textbooks, as well as with the aid of a micrometer. The material properties were provided by ANSYS Workbench’s built-in material library. The model was created using AutoCAD software (2022), consisting of an IGBT chip, diodes, a solder layer connecting the chip to the copper substrate, a copper plate, a ceramic layer, another solder layer, and a copper base [28]. Upon importing the IGBT model, the densities and thermal conductivities of various materials were set. With reference to Figure 1, the pertinent parameters are shown in Table 1 and Table 2.

### 2.3. Simulation Scenario Establishment

In order to make the simulation analysis experimental conditions similar to the normal working environment, this study simulates different solder layer materials under normal working conditions in terms of temperature, stress, and strain characteristics. To summarize the horizontal comparison, the following settings are established for this simulation process:

(1) IGBT chips are employed as the heat source, with an even heat distribution and disregarding thermal radiation’s impact on heat transfer. The chips’ thermal generation rate is assigned and its power loss is set at 100 watts. The heat generation rate is calculated as H = P/V = 8 × 10^9^ W/m^2^·K^−1^. Steady-state thermal simulation and static structural simulation analyses are conducted on the IGBT modules.
(2) This study focuses on the IGBT solder layer, intentionally neglecting external factors such as bonding wires to ensure the accuracy of experimental data. For ease of calculation, the analysis emphasizes the module’s temperature distribution and stress-strain, assuming material isotropy.
(3) In the simulation experiment, convection heat transfer is used to provide heat conduction and heat dissipation under normal working conditions. The equivalent convection heat transfer coefficient of the substrate is 3000 W/m^2^·K^−1^, the convection heat transfer coefficient around the substrate is 10 W/m^2^·K^−1^, and the heat flux around the IGBT chip is 1500 W/m^2^.
(4) The accuracy and quality of grid partitioning directly affect the results of subsequent finite element calculations. This paper uses high-precision meshing to break down the IGBT module into smaller, more manageable parts for numerical analysis and simulation.

### 2.4. Analysis of the Thermal Characteristics of IGBT Modules

**Figure 2:**
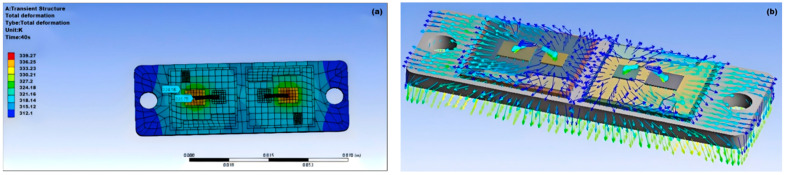
Thermal characteristics of the IGBT module: (**a**) IGBT temperature distribution; (**b**) IGBT heat flow direction.

A steady-state thermal analysis of the IGBT module loaded with 92.5Pb5Sn2.5Ag solder material is conducted. The temperature distribution of the IGBT module is shown in Figure 2a, and the heat flow direction is indicated in Figure 2b.

The simulation results show that the temperature distribution of the IGBT chip has distinct temperature differences in various areas, with the highest temperature at the center of the chip being 66.27 °C and the lowest temperature at the edges of the module being 39.1 °C. According to the thermal flow direction shown in Figure 2, the heat transfer between the internal structures of the module is from top to bottom, from the center of the chip to other areas, and the two chips influence each other.

## 3. Comparison of Thermal-Mechanical Coupling Analysis Results of Different Solders

### 3.1. Thermo-Mechanical Coupling Analysis of Nanosilver Solder

**Figure 3:**
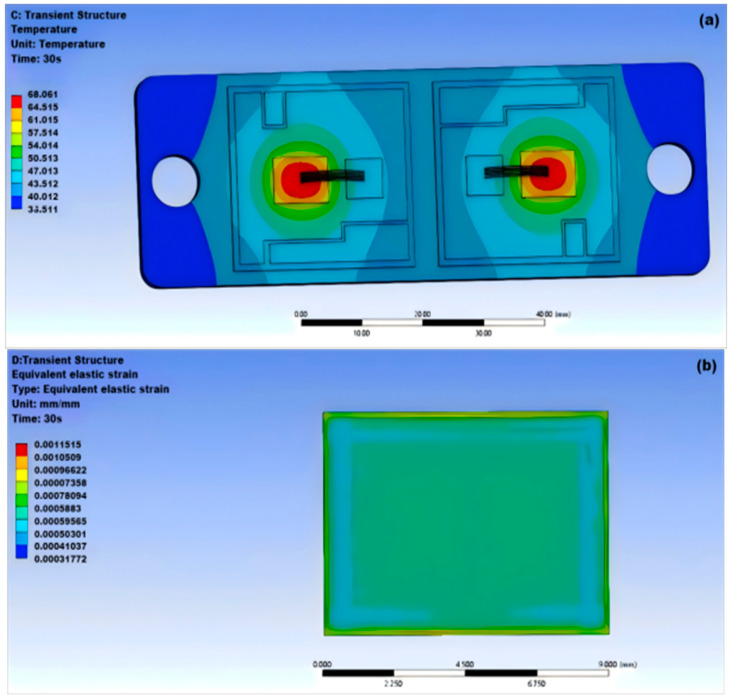

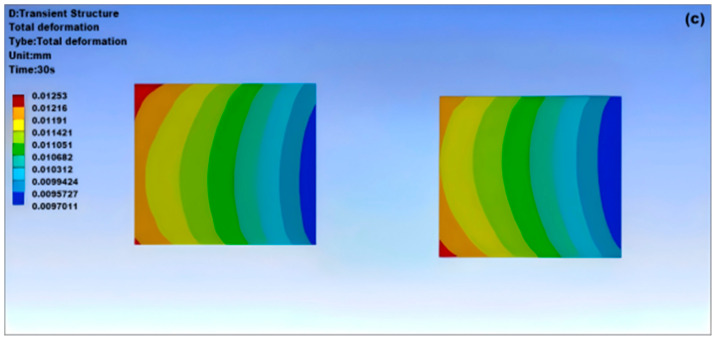
Thermal-mechanical coupling nanosilver solder: (**a**) thermal stability nanosilver solder; (**b**) equivalent elastic strain nanosilver solder; (**c**) surface deformation nanosilver solder.

Nanosilver solder is a type of welding material composed of nanosilver particles and several additives. It has good electrical and thermal conductivity, making it suitable for the production and assembly of electronic devices. As shown in Figure 3a, the highest temperature appears at the center of the solder layer at 68.016 °C, while the lowest temperature at the edge is 36.511 °C; as shown in Figure 3b,c, the module deformation amount is small and changes uniformly.

### 3.2. Thermo–Mechanical Coupling Analysis of SnPbAg Solder

**Figure 4:**
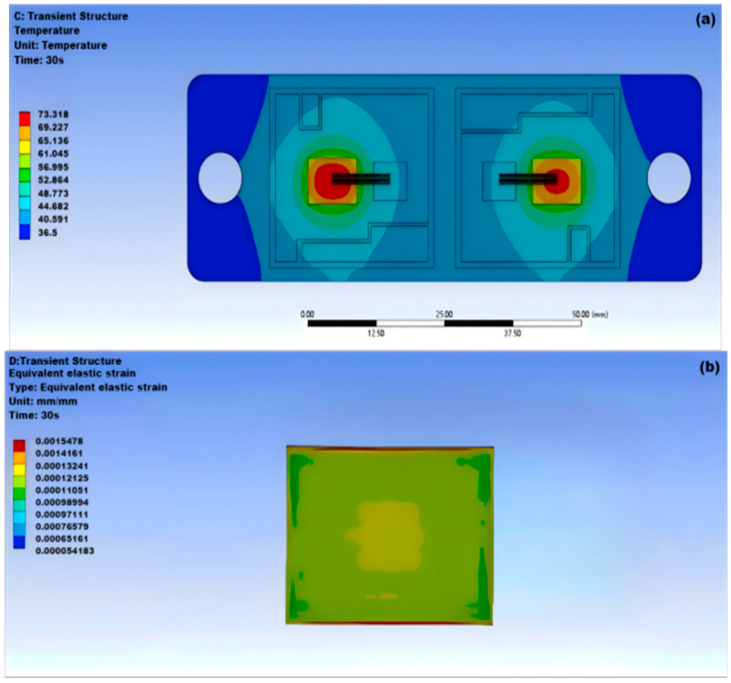

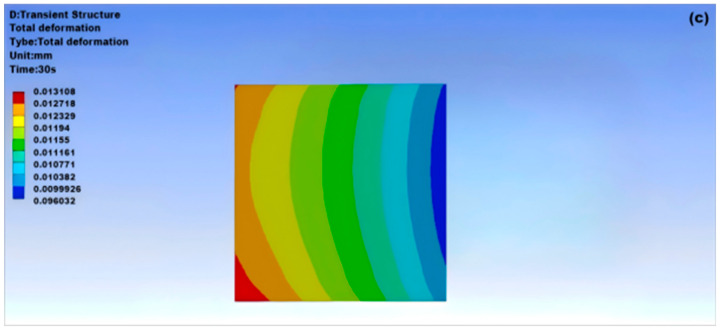
Thermal–mechanical coupling of SnPbAg solder: (**a**) thermal stability of SnPbAg solder; (**b**) equivalent elastic strain of SnPbAg solder; (**c**) surface deformation of SnPbAg solder.

SnPbAg solder is an alloy solder whose main components are tin (Sn), lead (Pb), and silver (Ag). As shown in Figure 4a, the maximum temperature of the module appears at the center of the solder layer at 73.318 °C, while the minimum temperature on both sides of the module is 36.5 °C; as shown in Figure 4b,c, the deformation of the module is considerable and varies unevenly.

### 3.3. Thermo–Mechanical Coupling Analysis of SnCu0.7 Solder

**Figure 5:**
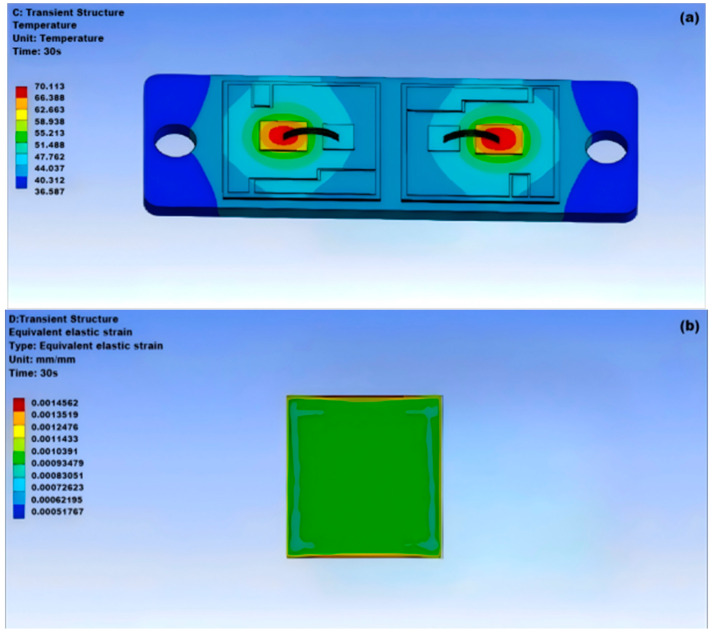

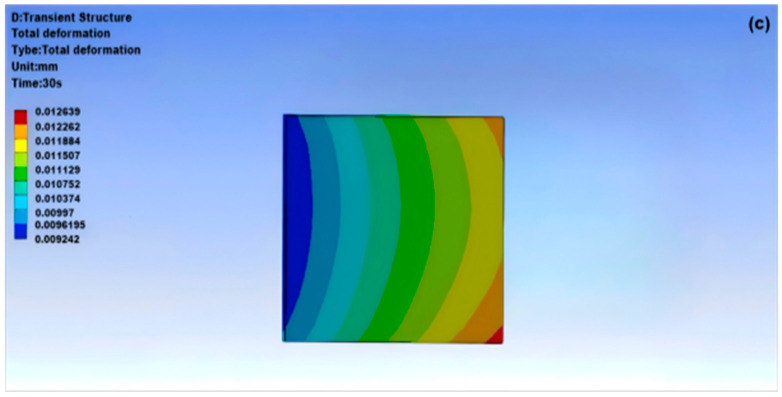
SnCu0.7 solder thermal–mechanical coupling: (**a**) SnCu0.7 thermal stability; (**b**) SnCu0.7 equivalent elastic strain; (**c**) SnCu0.7 solder surface deformation amount.

SnCu0.7 solder is a welding material composed of tin (Sn), copper (Cu), and a small amount of other elements. It is a lead-free solder material made from high-purity refined tin and advanced smelting processes. Compared to traditional lead-containing soldering materials, SnCu0.7 solder does not produce harmful lead pollution, which helps reduce environmental contamination. As shown in Figure 5a, the maximum temperature at the center of the solder layer is 70.113 °C, while the minimum temperature on both sides of the module is 36.587 °C; as indicated in Figure 5b,c, the deformation is minimal and uniform.

### 3.4. Thermo–Mechanical Coupling Analysis of Sn63Pb37 Solder

**Figure 6:**
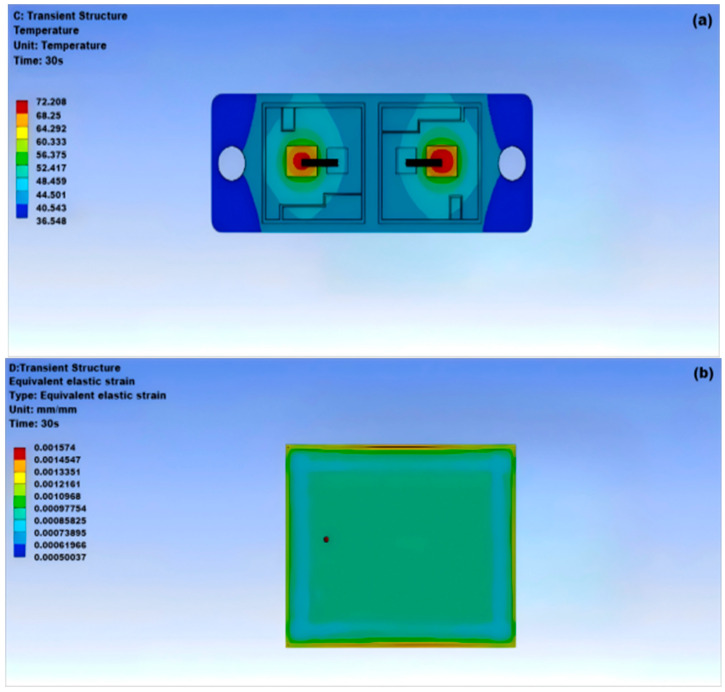

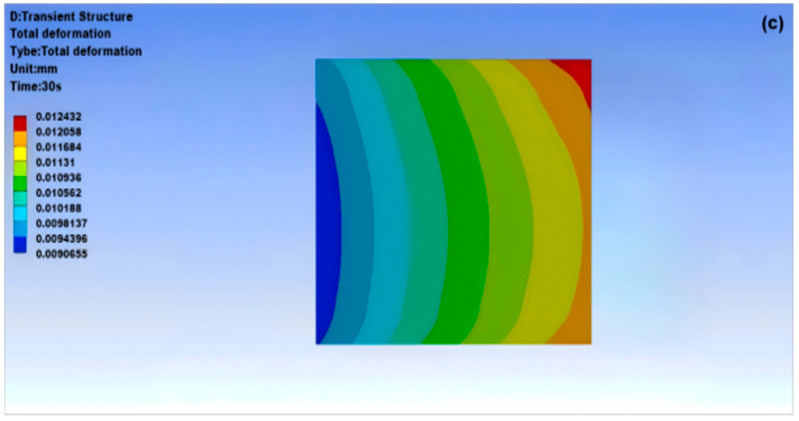
Thermal–mechanical coupling of Sn63Pb37: (**a**) thermal stability of Sn63Pb37; (**b**) equivalent elastic strain of Sn63Pb37; (**c**) surface deformation of Sn63Pb37 solder.

Sn63Pb37 solder is a eutectic solder composed of 63% Snand 37% Pb, featuring many excellent properties, a melting point of approximately 183 °C, and a density of about 8.40 g/cm^3^. As shown in Figure 6a, the highest temperature at the center of the solder layer is 72.208 °C, while the lowest temperature at the two wings is 36.584 °C; as illustrated in Figure 6b,c, the module undergoes significant deformation, but the changes are uniform.

### 3.5. Thermal–Mechanical Coupling Analysis of SnAgCu Solder

**Figure 7:**
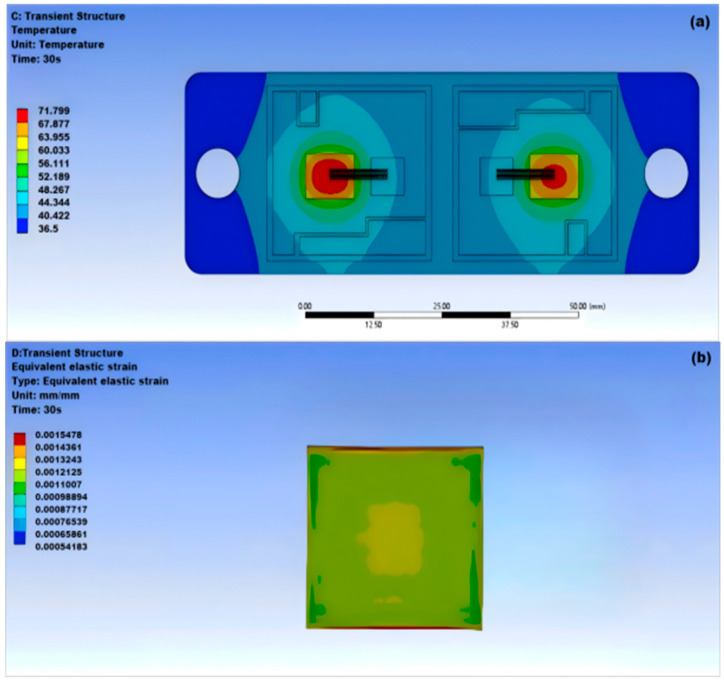

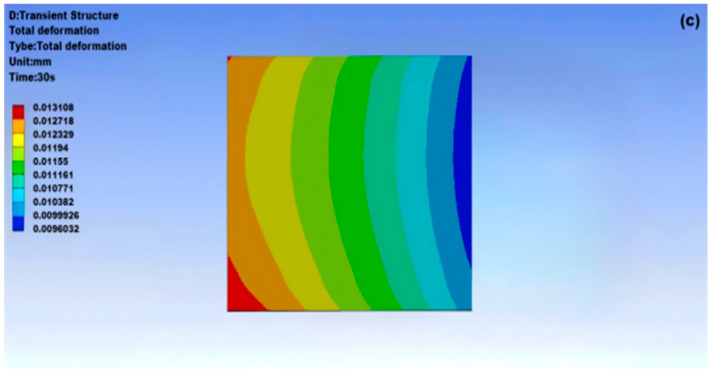
Thermal–mechanical coupling of SnAgCu solder: (**a**) thermal stability of SnAgCu solder; (**b**) equivalence of SnAgCu solder; (**c**) surface deformation of SnAgCu solder.

SnAgCu solder, also known as copper–nickel–silver solder or lead-free SnAgCu-based solder, is an alloy solder primarily composed of tin (Sn), silver (Ag), and copper (Cu). As shown in Figure 7a, the highest temperature at the center of the solder layer in the module is 71.799 °C, while the lowest temperature on both sides is 36.5 °C; as shown in Figure 7b,c, the module has significant and uneven deformation.

Based on the temperature distribution map of the five different solder materials mentioned above, it can be concluded that under normal operating conditions, the highest temperature of the IGBT module is at the center of the chip, while the lowest temperature is at the edges on both sides of the module [29,30,31]. As shown in Table 3, when comparing the temperature and equivalent stress-strain magnitude of the five solder materials, it is evident that the nanosilver solder has the best overall performance. The highest temperature of the module is the lowest among the five solders, which is due to the good heat dissipation capability of the silver element. Solder materials containing lead have a higher temperature compared to lead-free solder and have poorer heat dissipation capabilities.

Analyzing the equivalent elastic strain diagram, it can be seen that the stress distribution at the edge of the solder layer is uneven, while the stress variation in the center part of the solder layer is relatively smooth. The equivalent elastic strain and total deformation amount of different solder materials reflect their resistance to deformation under normal working conditions. Among them, the nanosilver solder experiences greater equivalent stress, but has smaller elastic strain, indicating strong deformation resistance. Additionally, among the other four traditional solder materials, Sn63Pb37 solder has the smallest total deformation of 0.012432 mm, outperforming other solder materials. The magnitude of thermal deformation is a reflection of the stress-strain results and is also caused by the differences in thermal expansion coefficients between each layer of materials, with the maximum deformation occurring at the region of the solder layer edge near the center of the module. The internal stress distribution of SnCu0.7 solder and nanosilver solder is relatively uniform, resulting in a more regular overall deformation.

## 4. The Impact of Solder Layer Damage on IGBT Modules

In power electronics systems, the reliability of the IGBT module is influenced by various factors. The solder layer is a critical connection part of the IGBT module and is also one of the most easily damaged structures [32,33]. The main reasons for solder layer damage in IGBT modules include the following aspects: (1) power cycling, which can cause cracks in the solder layer due to material fatigue; (2) solder layer fatigue, which can lead to breakage or delamination between the solder layer and the contact surface and increase the thermal resistance of the device, thereby accelerating overall device failure; and (3) differences in thermal expansion coefficients resulting in high stress, a direct cause of solder interface degradation. This stress can lead to deformation and stress concentration in the solder layer during temperature changes, thus increasing the risk of solder layer damage. The equivalent stress and equivalent elastic strain at the center of the solder layer are less than those at the edge position. Therefore, this simulation focuses on studying the temperature and deformation conditions at the edge position, keeping the solder material unchanged while only adjusting the form of solder layer damage, with other experimental conditions held constant to ensure the reliability of the experimental results.

### 4.1. Finite Element Analysis of IGBT Modules with Empty Cavity Damage

**Figure 8:**
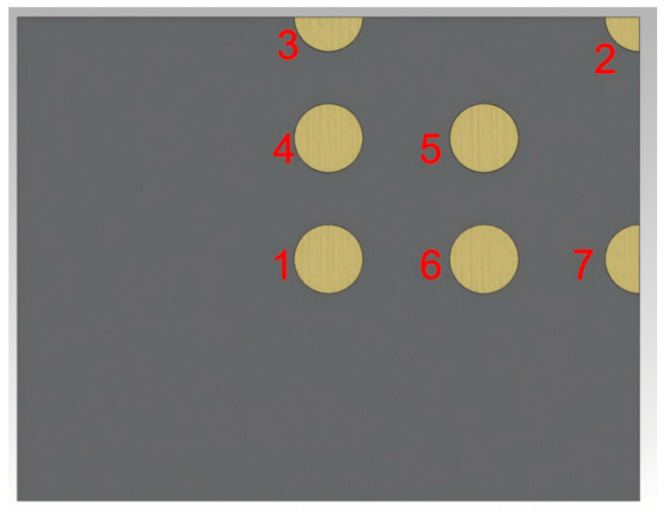
Positions of voids in the solder layer.

As shown in Figure 8, there are seven points with cavity damage in the IGBT. To better compare the temperature and stress changes caused by cavity damage, representative edge cavity positions 2 and 7, and central cavity positions 1 and 4, were selected for comparative analysis. These holes reflect the real defects that may appear within the solder layer under normal working conditions. Analyzing the temperature and stress changes in the module caused by void damage at different positions can help better protect and care for the IGBT modules by comparing the magnitude of the impacts.

#### 4.1.1. The Effect of Different Cavity Positions on Junction Temperature

**Figure 9:**
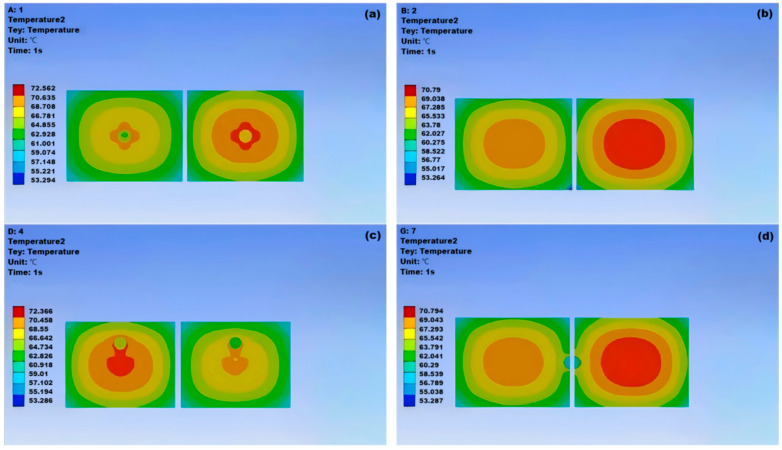
Temperature cloud map of solder layer at various cavity positions of IGBT: (**a**) cavity 1; (**b**) cavity 2; (**c**) cavity 4; (**d**) cavity 7.

As shown in Figure 9, void damage at different locations has a significant impact on the overall thermal behavior of the IGBT module. The presence of voids affects the direction of heat flow during module cooling, thus influencing the temperature distribution of the module. From the temperature distribution maps of the IGBT solder layer at various void locations, it can be observed that the position of the void causes the high-temperature center of the chip to shift, altering the normal heat dissipation path of the chip. Void 2 and void 7 are located at the edges of the module with small damage areas, resulting in junction temperatures about 70.79 °C lower than the other two void positions, which is close to the junction temperature of a normal module, indicating that such void positions have a minimal impact on the module’s heat dissipation capability. Void 3, similar to void 7, is also located at the edge of the module with a small damage area, with a junction temperature of approximately 70.63 °C. Void 1 and void 4 are located at the center of the module with a damage area of about 72.4 °C, indicating that void damage at these positions significantly reduces the module’s heat dissipation capability. Although void 5 and void 6 are not at the center, their damage areas are similar to those of void 1 and void 4, with a junction temperature of approximately 71.3 °C. Therefore, if void damage is unavoidable, efforts should be made to locate the voids at the edges with small damage areas to ensure the reliability of the module.

#### 4.1.2. The Effect of Different Cavity Locations on Stress

**Figure 10:**
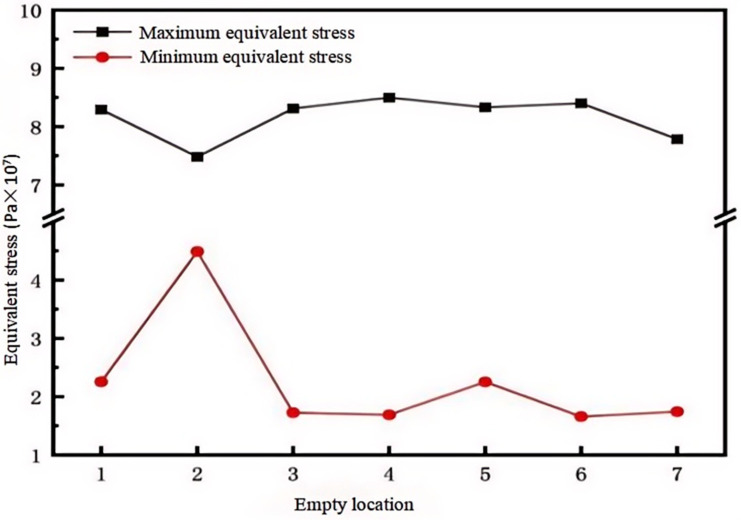
Equivalent stress of solder layer at different cavity locations.

As shown in Figure 10, the average equivalent stress at the location of cavity 2 is the highest—approximately 6 × 10^7^ Pa. The average equivalent stress is less than this at the locations of cavities 1, 4, and 7. This difference may be due to the fact that cavity 2 is located at the edge corner of the module. From the data in Table 4, it can be seen that the thermal strain at the edge positions of cavities 2 and 7 is less than that at the center positions of cavities 1 and 4, but cavities 1 and 4 show more regular deformation in the *x*-axis direction. Cavity 2 has a larger deformation compared to 7, which leads to the inference that the location of cavity 2, being at the edge corner, lacks constraints from the surrounding area, and thus experiences greater deformation.

### 4.2. The Impact of Crack Damage on IGBT Modules

The solder layer is a crucial component of the IGBT module, and cracks or damage in it can directly affect the module’s thermal performance and structural stability. Cracks in the solder layer can increase thermal resistance, leading to accelerated junction temperature rise. This damage disrupts the continuity of the solder layer, hindering heat transfer and causing heat to accumulate in localized areas, resulting in increased junction temperature. The rise in junction temperature further exacerbates thermal stress in the solder layer, creating a vicious cycle that can lead to cracks in the solder layer and device failure in severe cases. In addition, cracks in the solder layer also affect the stress distribution within the module [34,35,36]. Due to the different thermal expansion coefficients between layers, changes in temperature distribution within the module can induce shear or tensile stresses between layers. Cracks in the solder layer alter the pathways and distribution of stress transmission, causing stress concentration in certain areas and increasing the risk of module failure.

**Figure 11:**
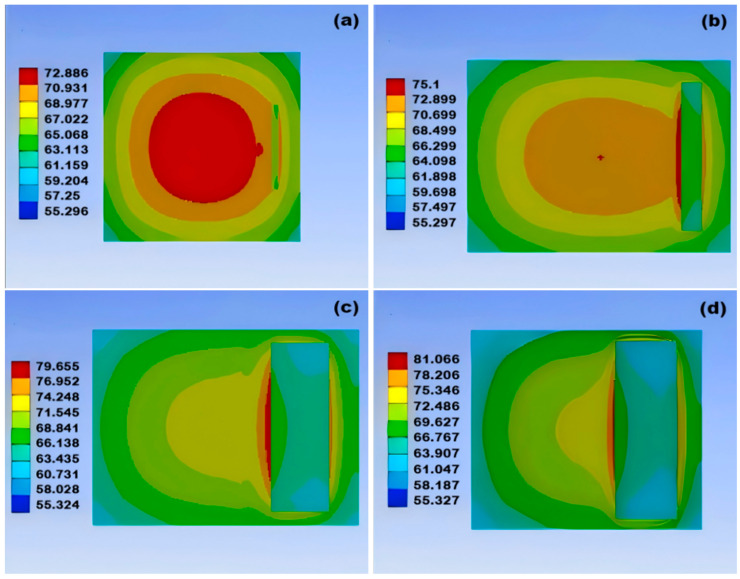
Cloud diagram of temperature changes of non-through holes as void size increases: (**a**) temperature change cloud diagram for 0.1 mm void; (**b**) temperature change cloud diagram for 0.7 mm void; (**c**) temperature change cloud diagram for 0.9 mm void; (**d**) temperature change cloud diagram for 1 mm void.

**Figure 12:**
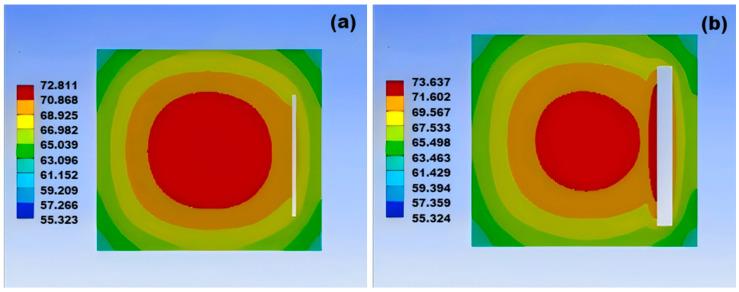

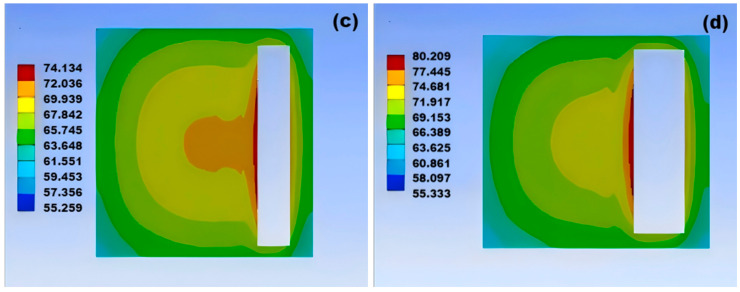
Temperature variation contour map of through-hole welding with increasing cavity size: (**a**) temperature variation contour map for 0.3 mm cavity; (**b**) temperature variation contour map for 0.5 mm cavity; (**c**) temperature variation contour map for 0.7 mm cavity; (**d**) temperature variation contour map for 1 mm cavity.

As shown in the temperature variation cloud charts of the damage module in Figure 11 and Figure 12, it can be concluded that, as the size of the crack increases, the highest temperature point of the solder layer continuously moves in the direction of the crack direction as the module temperature keeps rising, indicating that crack damage affects the heat dissipation capability of the module. Further comparison reveals that the temperature distribution of non-through cracks changes more rapidly, which indicates that the damage from through cracks is more destructive to the module than non-through damage.

**Figure 13:**
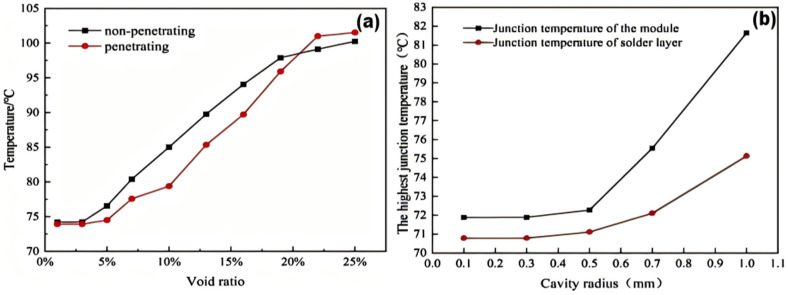
Variation of junction temperature and cavity radius trend: (**a**) junction temperature variation under non-through and through crack forms; (**b**) junction temperature variation with cavity radius under non-through and through conditions.

As shown in Figure 13a,b, when the porosity is at 5% or the cavity radius is below 0.5 mm, the temperature variation of the solder layer remains stable, indicating that the thermal behavior of the IGBT is not significantly affected. Therefore, the porosity of the IGBT module should ideally be controlled at 5% and the cavity radius below 0.5 mm. When the porosity varies in the range of 5% to 20%, the temperature of the IGBT module increases with the rise in porosity and cavity radius, which greatly affects the operational performance of the module.

#### The Effect of Crack Size on the Stress in the Solder Layer

**Figure 14:**
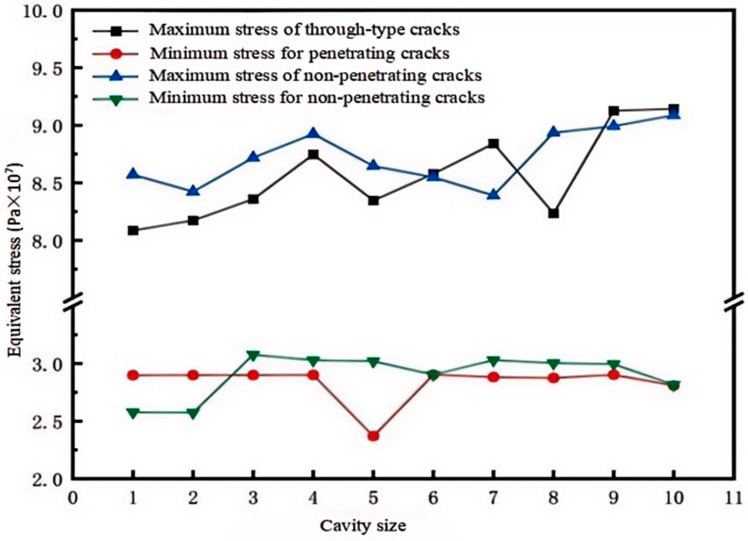
Equivalent stress of solder layer under different crack damage.

As shown in Figure 14, with the increase of damage, the overall stress shows a rising trend. The maximum stress fluctuations under the through cracks are significant, while the maximum stress of non-through cracks is relatively stable, and the overall stress is lower than that of through damage; moreover, the variation trend of the maximum and minimum stress for non-through cracks is more consistent compared to that of through cracks. It can be seen that the harm of non-through cracks to the overall structure of the module is less than that of through cracks.

## 5. Conclusions

This article utilizes ANSYS Workbench software to analyze and compare the thermal and mechanical properties of five types of solder materials, thereby revealing the variation patterns of thermal–mechanical interactions in IGBT solder materials. The goal is to optimize the design and process parameters of IGBTs, compare and evaluate different types of solder materials to select the most suitable solder material, improve the working efficiency and lifespan of IGBTs, and reduce the risk of failures caused by solder material failure to ensure their reliability. The following are the conclusions drawn from this article:

(1) According to the simulation results, under normal operating conditions, the highest temperature of the IGBT chip module is 66.27 °C at the center of the chip; the lowest temperature is 39.1 °C at the edges of the module on both sides.
(2) The maximum temperature of the nanosilver solder in thermal stability simulation analysis is 68.016 °C and the minimum temperature is 36.511 °C; it has the overall lowest temperature among the five solder materials.
(3) The equivalent stress experienced by the nanosilver solder is greater, but the elastic strain is smaller at 0.00115 mm, and the total deformation is 0.01253 mm.
(4) Cavity damage located at the edge has less impact on the junction temperature than that near the center of the solder layer. Cavity damage at the edge angle generates greater strain in terms of stress. For crack damage, non-through cracks transfer heat more quickly. When the cavity rate is 5% or the cavity radius is below 0.5 mm, the junction temperature of the solder layer remains relatively stable compared to the overall junction temperature, indicating that the thermal behavior of the IGBT is not significantly affected. Therefore, it is best to control the cavity rate of the IGBT module at 5% and the cavity radius below 0.5 mm. When the cavity rate varies between 5% and 20%, the temperature of the IGBT module increases with the rising cavity rate and cavity radius, greatly affecting the operational performance of the module.

## Figures and Tables

**Table 1:** The geometric dimensions of IGBT modules.

Module Structure Nomenclature	Length/mm	Width/mm	Thickness/mm
Chip	9	7	0.2
Diode	6	6	0.2
Chip solder layer	9	7	0.12
DBC on/Lower copper layer	30	28	0.3
DBC ceramic layer	30	28	0.38
Base solder layer	30	28	0.12
Copper substrate	9	32	3

**Table 2:** Material parameters of IGBT modules.

Material Parameters	Density kg/m^3^	Thermal Conductivity W/(m·k)	Thermal Expansion Coefficient 10^−6^k^−1^	Poisson’s Ratio	Specific Heat Capacity J/(kg·k)	Young’s Modulus
Cu	8600	390	17	0.37	390	110,000
SnAgCu	7300	54	25	0.4	230	34,300
AIN	3400	1700	4.5	0.22	710	3200
AI	2690	119	2.99	0.28	700	167,000
Nano silver	8500	160	19.5	0.25	235	50,000
92.5Pb5Sn2.5Ag	11,000	35.8	29	0.35	170	24,700
Air	1.09	2.733				
Sn63Pb37	8400	51	25	0.189	150	28,000
SnCu0.7	7310	80	23	0.37	220	30,000

**Table 3:** Simulation analysis data of five types of solder materials.

Solder Material	Maximum Temperature °C	Minimum Temperature °C	Equivalent Elastic Strain mm\mm	Total Deformation mm
Nanosilver	68.016	36.511	0.00115	0.01253
SnPbAg	73.318	36.5	0.00171	0.01284
SnCu0.7	70.799	36.587	0.00146	0.012639
Sn63Pb37	72.208	36.584	0.00157	0.012432
SnAgCu	71.799	36.5	0.00155	0.013108

**Table 4:** Thermal strain and deformation of solder layer corresponding to different cavity loss locations.

Location Number	Thermal Strain (m/m)	*X*-Axis Directional Deformation Amount Max (m)	*X*-Axis Directional Deformation Amount Min (m)	Total Deformation (m)
1	1.2642	1.2964 × 10^−6^	−1.2474 × 10^−6^	4.7316 × 10^−6^
2	1.2198	1.3027 × 10^−6^	−1.2539 × 10^−6^	4.7292 × 10^−6^
3	1.2198	1.3025 × 10^−6^	−1.2535 × 10^−6^	4.7286 × 10^−6^
4	1.2592	1.3013 × 10^−6^	−1.2527 × 10^−6^	4.7287 × 10^−6^
5	1.2532	1.3034 × 10^−6^	−1.2502 × 10^−6^	4.7277 × 10^−6^
6	1.2622	1.2934 × 10^−6^	−1.2444 × 10^−6^	4.7313 × 10^−6^
7	1.2198	1.3024 × 10^−6^	−1.2532 × 10^−6^	4.7289 × 10^−6^

## Data Availability

The original contributions presented in the study are included in the article; further inquiries can be directed to the corresponding author.
